# Videos/Film

**DOI:** 10.1155/1990/75967

**Published:** 1990


VF001 PANCREATODUODENECTOMY WITH PYLORIC PRESERVATION

G. Tiberio, S.M. Giulini, L. Cangiotti, N. Portolani

Department of Surgery - BRESCIA - Italy

The film illustrates the pancreatoduodenectomy operation carried out on a 60 year-old patient, affected by an ampullary adenocarcinoma. The technique adopted involves the removal of the duodenumpancreatic section with pyloric preservation according to the technique proposed by Traverso and Longmire with the aim of simplifying the surgical procedure and of improving the post-operative nutritional state of the patient. Gastric preservation doesn't preclude the possibility of a radical lymphadenectomy, the perigastric and splenic nodes being only on exception site of metastasis in such pathology.

The first step is the mobilization of the hepatic flexure of the colon and of the second duodenal portion; the isolation of the distal choledoch duct and of the upper mesenteric vein in its retropancreatic section allows the completion of the mobilization of the pancreatic head and of the third and fourth duodenal portions. The technical shrewdness that the operation foresees is seen in the film: the accurate saving of the innervation and of the vascularization of the proximal duodenal section, realised in the specific case with the respect of the gastroduodenal artery and of both of the gastric vascular arches, the supramesocolic transposition of a loop of jejunum dorsal to the mesenteric vessels in the bed of the resected duodenum, the accurate surgical technique turned towards preventing the formation of haematic and lymphatic gatherings which can induce a transitory postoperative gastric paresis. The pancreatic and biliary anastomoses are subsequently carried out by means of a termino-lateral technique in a two layer fashion in re-absorbable synthetic material; the end-to-side jejunostomy, carried out with continuous stitches results as technically easy and, even though it is a short distance from the pylorus, does not compromise its function. The accurate perianastomotic drainage completes the operation, resulting free of complications.

VF002 PYLORUS - PRESERVING DUODENOCEPHALOPANCREASECTOMY FOR AMPULLARY CARCINOMA

G. Romeo,
G. Catania, F. Basile, F. Cardì, G. Azzarello,
G. Beneventano, M. Migliore, A. Bucceri
Surgical Pathology 2nd - Catania University
Faculty of Medicine, Catania, Italy

Duodenocephalopancreasectomy represents the only change for cure of ampullary and periampullary tumors. But digestive and nutritional sequelae have been registred after the standard pancreaticoduodenectomy ( Whipple procedure ).
Pylorus and gastric preservation, according to Traverso - Longmire technique, seems not to riduce the oncologic radicality, has the advantage of a shorter operative time and a simpler esecution and it showed assure less digestive postoperative sequelae with better nutritional status of patients.
The AA. present a case of ampullary carcinoma treated by pylorus-preserving duodenopancreasectomy; the postoperative radiologic and manometric studies have demonstrated a good pyloric function with phisiological empting of the stomach.

### VF003 PARTIAL DUODENOPANKREATECTOMY WITH RADICAL LYMPHADENECTOMY

D. Henne-Bruns, B. Kremer
Dep. of Surgery, Univ. of Hamburg
FRG

The video demonstrates the preoperative diagnostic procedures like ERCP, endosonography, CT-scan and angiography in patients with distal bile duct carcinomas, carcinomas of the head of the pancreas, tumors of the papilla and duodenum. The following operation shows a partial duodeno-pancreatectomy with extended retroperitoneal lymphadenectomy. The operation starts with the dissection of the ventral surface of the right kidney. Afterwards the caval vein and the confluence region of the renal veins are exposed. The mobilized tissue from these structures remains at the duodenum, to be removed later on with the resectate.
After cholecystectomy the common bile duct is transsected. The hepatic artery is isolated and freed from lymphatic tissue up to the celiac trunc. Afterwards the portal vein is isolated. The preparation continues with the lymphadenectomy between the vena cava and the aorta proximally and distally of the left renal vein. Thereby the superior mesenteric artery is isolated and freed from lymphatic tissue.
The stomach is transsected after skleletonization. After skletonization of the first jejunal loop and distal duodenum, the resectate is transsected from the superior mesenteric vein and removed. The reconstruction is performed with two jejunal loops. The first loop drains the pancreas and the bile duct, the second loop drains the stomach.

VF004 DISSECTION OF PERIAORTIC LYMPH NODES FOLLOWED BY CUT OF THE LEFT RENAL VEIN IN EXTENDED PANCREATODUODENECTOMY

T.Nagakawa, K.Ueno, T.Ohta,

H.Kobayashi, Y.Nakano, T.Nakamura,

M.Kadoya, M.Kayahara, I.Konishi,

T.Matsumoto, I.Miyazaki, Kanazawa

University, Kanazawa, Japan

In order to improve the surgical results of carcinoma of the pancreatic cancer, since 1973, we have prefomed a radical operation including resection of the pancreatic nerve plexus, dissection of the lymph nodes around the pancreas and the abdominal aorta, and resection of the portal vein. Recently we performed dissection of pancreatic lymph nodes followed by cut of the left renal artery as one of more extensive surgery. So, this precedure is presented in film.

With these efforts 8 patients survived for 3 years or more, and the 5-year survival rates in patients with cancer of the pancreatic head who tolerated macroscopic curative resection became 36.5%. Lymph node metastasis was negative in 6 cases (35.3%) and positive in 11 (64.7%). The positive metastatic cases consisted of 7 cases with $n_1$, 3 with $n_2$ and one with $n_3$. While the negative metastatic group showed a one-year survival rate of 100%, a 2 to 3-year survival rate of 83.3% and a 3 to 4-year survival rate of 66.7%, 27.3% of the positive patients survived one year, only one, 2 years or more and 9.1%, 5 years. These findings show that more extensive surgery is necessary for the treatment of the pancreatic cancer.

References:

Nagakawa T, et al. Jap. J. Surg. 1989; 19: 5.

### VF005 PANCREATODUODENECTOMY FOR PERI-AMPULLARY CANCER - OUR MODIFIED PROCEDURE

T.Suzuki, Y.Hamanaka, A.Kawamura, Y.Shinagawa, M.Nishikawa,
Department of Surgery II , Yamaguchi University, Ube, Yamaguchi, JAPAN

The procedure of our modified pylorus-preserving pancreatoduodenectomy is presented on a 66 year old male with cancer of the ampulla of Vater. The duodenum is resected with the pancreatic head tissue, but the jejunum is preserved completely : the oral dissected duodenal margin is 3 to 4 cm distal to the pyloric ring, and the anal duodenal margin is the fourth portion of the duodenum. Dissection of the lymph nodes along the superior mesenteric artery, the hepatic artery and the hepatoduodenal ligament is made; but the right and left gastric arteries as well as the hepatogastric ligament are all preserved. The Billroth I type of reconstruction is adopted. First of all,an end-to-end duodenoduodenostomy is performed. Then an end-to-side pancreatojejunostomy is made 10 cm anal to this duodenoduodenostomy; and 5 cm anal to the pancreatojejunostomy, an end-to-side hepatidochojejunostomy is added. No Braun anastomosis is made. The stent tube inserted into the main pancreatic duct is drawn out from the anterior wall of the stomach. After this type of resection and reconstruction, regulation of the pH and enzymatic digestion of diet are thought to be maintained more physiologically than after the hitherto reported any types of procedures, because food or gastric juice passing through the pylorus immediately stimulates the duodenum and the proximal jejunum to release various gut hormones, resulting in effective stimulation of bile and pancreatic juice secretions, with which the food is mixed promptly and satisfactorily. Since 1981, we have performed this modified pancreatoduodenectomy. The postoperative course of the patients has been uneventful except for a transient delayed gastric emptying. No marginal ulcer has developed.

Reference: Suzuki,T.: World J Surg 1988: 12: 645-650

VF006

### ISOLATED PANCREATECTOMY FOR DUCTAL CARCINOMA OF THE HEAD OF THE PANCREAS

H. Mimura, K. Hamazaki, H. Tsuge, M. Mori
Okayama University Medical School, Japan

To improve the poor survival after resection of ductal carcinoma of the head of the pancreas, the most frequent evidence of it's recurrence in the liver and local retroperitoneum should be controlled. The major cause of the early liver metastasis, we assume, might be due to migration of the carcinoma cells into the portal vein during the operative manipulation, while the local retroperitoneal recurrence would be caused by incomplete resection of the retroperitoneal tissue. Accordingly, we devised a new method of non-touch pancreatic resection -"Isolated Pancreatectomy". This is pancreatectomy performed under occlusion of the blood flow around the head of the pancreas or tumor, achieved by clamping of the portal vein, splenic vein and superior mesenteric vein, with preceeding ligation of the gastroduodenal artery and inferior pancreatic artery. These vessels are removed together with the pancreatic tumor, thus giving a wide surgical field facilitating extensive removal of the retroperitoneal lymph nodes and nerve plexus. During this procedure for retroperitoneal skeletonization under dividing of the portal vein for one or two hours, a catheter bypass of the portal flow into the femoral vein is effective for avoiding portal congestion. Reconstruction of the portal vein is always necessary.

We have used this method in nine patients over the past two years, and both hepatic and local recurrence has been reduced.

The film shows the details of this method.

### VF007 ENUCLEATION AND "INTERMEDIATE PANCREATECTOMY" IN TWO CASES OF INSULINOMAS OF THE PANCREAS

Serio G. ,Iacono C.,Modena S.,Trivisone M.,Dagradi A.
Clinica Chirurgica - University of Verona - Verona, Italy.

The radical therapy of insulinomas is exclusively surgical,consisting either in enucleation of the tumor or in pancreatic resection.

The relatively less frequent localization in the head and neck gives rise to problems of technical choice, especially if the tumor is two centimeters in diameter or larger.Tumorectomies of the neck are associated with high risk of injury to the main pancreatic duct; on the other hand a subtotal pancreatic resection entails the risk of post-operative diabetes (Yasugi et al.1976). As for the insulinomas of the head of the pancreas, a Whipple operation would appear unjustified in most cases,owing to the fact that 80% to 90%of such tumors are benign (Stefanini et al. 1974). Once again the simple tumorectomy is not free of risk of inadvertent damage to the pancreatic duct, to the intrapancreatic portion of the common bile duct or to the mesenteric vessels. The two cases we propose offer two different technical solutions to avoid extensive parenchymal demolition.

Case 1 : Male,aged 50, obese, with sympoms (fatigue,tremors,fasting perspiration) lasting for 3 years. Fasting blood sugar was very low and blood insulin high .

Repeated provocative tests were positive for hyperinsulinism. Pre-operative US and CT scans failed to demonstrate a pancreatic tumor. After the failure of a three-months course of medical treatment, angiography demonstrated a 2 cm. hypervascularized area in the head of the pancreas, fed trought a hepatic artery originating from the superior mesenteric artery; the intraoperative US scan showed a single tumor lying adjacent to the main pancreatic duct, to the common bile duct and to the portal vein. The tumor was enucleated, and a transduodenal pancreatography showed no leak from the main duct. The post-operative period was complicated by a low-output pancreatic fistula, wich healed on day 15 after treatment with somatostatin, H2-blockers and parenteral nutrition. Normalization of glycemia and insulinemia, and discharge on day 23.

Case 2: Female,aged 74,with symptoms lasting for 9 years; after 7 years of relative control under medical treatment, symptoms relapsed with fasting blood sugar values of 30-50 mg/dl and hyperinsulinism. US and CT scans showed a 18 mm. solid tumor of the pancreatic neck. The intraoperative US scan showed a single tumor occupyng the full thickness of the neck of the pancreas. We performed a segmental resection of the tumoral area with section lines 1 cm. to the right and left of the tumoral margin (intermediate pancreatectomy). After suture of the cephalic stump we performed a left end-to-end Roux-en-Y pancreojejunostomy.The post-operative period was uneventful. There was gradual normalization of glycemia,glycosuria and insulinemia. The patient was discharged on day 15.

References :

1) Yasugi H.,Mizumoto R.,Sakurai H.,Honjo I.: Am. J.Surg. 1976:132,377.
2) Stefanini P.,Carboni M.,Patresi N.,Besali A.,: Surgery 1974:75,597.

**VF008 DUODENAL SOMATOSTATINOMA ASSOCIATED WITH VON RECKLINGHAUSEN'S DISEASE: A RARE CLINICAL PATHOLOGICAL ENTITY.**

G.P.Spina, E.Opocher, G.Gagliano, R.Santambrogio, F.Galeotti, G.Rubis Passoni, G.Viale. San Paolo Institute of Biomedical Science - University of Milan - Italy

The association between Von Recklinghausen's disease and somatostatinoma is an uncommon but not surprising event. Both diseases are due to an endocrine tissue disorder and have a common embryological origin.
The videotape starts showing the case of a young woman of 38 who has borne the signs of Von Recklinghausen's disease since birth. She was admitted to our Department with a ten month history of mild jaundice and moderately increased cholestasis indexes. The imaging examinations, even if they confirmed a extrahepatic cholestasis due to an obstacle at the level of the papilla of Vater, did not propose a conclusive diagnosis of the lesion.
The central part of the videotape shows the technical phases of the surgical operation: the explorative phase that showed the presence of a well-circumscribed neoformation on the level of the Vater region; the dissecting phase that was the removal of duodeno-pancreatic block with the pylorus preservation and the reconstruction phase according to the technique proposed by Traverso-Longmire.
The video tape finishes showing the histological and immuno-histochemical analyses of the lesion: it was a neoplastic proliferation of epithelial cells with numerous granules of a neuro-secretory type containing somatostatin.
Finally, general and prognostic characteristics of this rare association are discussed.

**VF009** DOUBLE CENTRAL CHOLANGIO-JEJUNOSTOY AFTER MULTIPLE OPERATIONS BY IATRO-JENIC LESION OF THE COMMON DUCT

E. MORENO; I. GARCIA; P. RICO; R. GOMEZ; J. SEOANE; J. BERCEDO; H. BONET

Hospital 12 de Octubre. Madrid. Spain

A lady was operated firstly and gallbladder was removed, section of the common duct occurred. The patient was treated by surgery four times (end-to-end anastomosis, choledocho-duodenostomy, hepatico-jejunostomy and double hepatico-jejunostomy with stent) but cholangitis continue and the patient was progressively deteriorated. The operation consist in a new dissection of both hepatic ducts, removal of scar and fibrous tissue doing two specily wider separated hepatico-jejunostomy. The patient cured absolutely without cholangitis during the last three years.

### VF010 INTRAHEPATIC CHOLANGIOJEJUNOSTOMY

H. Bekada, O. Benhadid, S. Metehri
B. Mentouri, Algiers, Algeria

A 30 year old man was being operated on for biliary stenosis after hepatic ducts injury.

He had jaundice 5 days after cholecystectomy was performed for cholecystitis 2 months previously in a country hospital.

A transhepatic cholangiography showed a type 111 stenosis of the bile ducts.

At laparotomy the stenosis was located in the hilum and preoperative cholangiography showed a type IV stenosis. A bilateral cholangiojejunostomy was performed with a Roux en Y loop.

Intrahepatic left cholangiojejunostomy was performed side to side and right cholangiojejunostomy was performed end to side with a transanastomotic tube because of an injured anterior wall of the right hepatic duct. A post operative cholangiography showed good functional anastomosis on the 10th day and jaundice disappeared completely after 20 days.

18 months later the patient is doing very well without any jaundice or complaint.

VF011 RESECTION OF CENTRAL BILE DUCT CARCINOMA AND RECONSTRUCTION BY CHOLANGIO-DUODENAL INTERPOSITION OF A JEJUNAL SEGMENT.

B. Kremer, D. Henne-Bruns
Dep. of Surgery, Univ. of Hamburg
FRG

The video demonstrates the preoperative diagnostic procedures like ERCP, endosonography, CT-scan and angiography to evaluate the extent of tumor growth in central bile duct carcinomas.
The afterwards demonstrated operation was performed in a patient with a central bile duct carcinoma Typ II according to the classification of Bismuth. The video shows the preparation of the hepato-duodenal ligament including a radical lymphadenectomy of this region. The hepatic artery and portal vein are thereby exposed and the common bile duct as well as the confluence region of central bile ducts is resected.
The reconstruction is not as usual performed in a Roux-en-Y technique but by a cholangio-duodenal interposition of a 20 cm jejunal segment.
The advantage of this technique is demonstrated by the follow-up endoscopy. It is shown, that the in this case 5 cholangio-jejunal anastomoses can be identified endoscopically. This means, that a recurrent stenosis of this region could be treated endoscopically for example by laser resection.

### VFO12 COMBINED SURGICAL AND ENDOSCOPIC APPROACH OF THE ACUTE COLANGITIS.

V.Scortecci, F.L.Dassi, C.Crosta, A.Cerizzi
L.Vicentini, M.Nosotti, A.M.Taschieri.

Institute of General and Thoracic Surgery
Milan, Italy

In the past the therapy of acute colangitis was loaded with high morbidity and mortality.
Emergency surgical drainage and wide spectrum antibiotics were the only therapeutic resorts.
In the last ten years a number of auxiliary procedures improved the diagnostic capacity and reduced morbidity and operative mortality.
Today the basilar goal of ensuring the patency of the biliary tree and of sterilizing the bile itself can be reached by percutaneous or retrograde endoscopic drainage which also allow the administration of antibiotics in the infected areas.
These techniques allow sometime, by endoscopic surgical procedures such the sphincterotomy, to ensure the definitive solution of the clinical problem; if this needs a true surgical approach, this can be delayed to a later time, when PTC and ERCP have obtained the control of the infection and of the biliary stasis.
The videotape offers some clinical examples of the approaches that we think advisable in the different patients.

<u>References:</u>

C.Crosta, A.M.Taschieri Chir.Arch.Trim.1986; 10: 143
M.E.Pessa, F.Hawkins Ann.Surg.1987; 205: 389
Wan-Yee Lau, Kin Kee Chong Ann.Surg.1987; 206: 142

VFO13 PERCUTANEOUS APPROACH TO THE BILIARY TRACT

R.Murai, A.Kusuyama, K.Watanabe,
F.Hashiguchi, H.Ando, K.Itsubo
Jikei University School of Medicine,
Tokyo, JAPAN

Nowadays Percutaneous Transhepatic Biliary Drainage (PTBD) is performed not only for reducing jaundice but also preoperative diagnosis by Percutaneous Transhepatic Cholangio Scopy (PTCS) or percutaneous stenting.

We present our percutaneous approach to the Biliary Tract for the patients with obstructive jaundice by Video.

Firstly obstructive jaundice is diagnosed by Ultrasonography and should be drained as soon as possible. We prefer PTBD to Endoscopic Stenting because we need the fine information of the hepatic bile duct for surgical operations.

Secondly the PTBD tract is dilated and biopsies are taken under X-TV.

Thirdly a Cholangioscope is inserted through the dilated PTBD fistula. We can observe the stricture site and take biopsy under direct vision. Consequently we decide the resection point and operative method.

In the case of non-resectable or recurrent tumors, we place a double mushroom stent across the stricture of the bile duct.

Since 1986 we have performed PTBD in 92 cases and 84 of these cases were successful (91.3%). Percutaneous stenting was tried in 14 cases out of 92 (15.2%) and 12 cases were successful (85.7%).

These procedures take 2 or 3 weeks, but usually reducing of icterus takes more than 2 weeks.

Obviously the advantage of the percutaneous approach is to get details of the lesion. Most of the Bile Duct Cancers have scirrhous invasion pathologically. Therefore to know the microscopic findings of the bile duct near the tumor is particularly important for the curative operations.

### VFO14 COMBINED SURGICAL AND ENDOSCOPIC APPROACH IN COMPLICATED BILIARY LITHIASIS

VIANINI R. - CACIOLI D. - TOTI G.L.
FIORI G.C. - FERRANTE F. - MEGEVAND J. - GALEONE M.
SURGICAL DEPT. SESTO SAN GIOVANNI HOSPITAL - MILAN

The morbidity & mortality rate in biliary lithiasis depend on a variety of factors--age, the necessity of emergency surgery & its associated complications. The literature reveals that cholelithiasis is complicated by acute cholecystitis in 1.6%, empyema in 1.4%, complications rising to 4.3% & 8.7% in patients over 60. Regardless of age late surgery for acute cholecystitis reveals a 5% mortality rate & a doubling of post-op stay (20.1 vs 10.9 days). Yet emergency surgery in this field shows a 94% rate of positive bile cultures (B.C.) with consequent 41% wound infection (W.I.)rate & an 18% rate of septicemia. We find in elective surgery 25% of positive B.C. with consequent W.I. of 18% & septicemia in 6% of all cases. We divide the elective patients into two subgroups: A) simple cholecystectomy (12% of positive B.C. with 10% W.I. & 1% septicemia). B) cholecystectomy + choledochotomy (46% of positive B.C. with 31% W.I. & 12% septicemia). We deduce that surgical therapy on a complicated biliary lithiasis, especially in elderly patients, should be performed early, & without surgical penetration into the common bile duct or into the duodenum. This rationale constitutes the basis of our proposed technique of surgical & perioperative endoscopic sphincterotomy to resolve complicated biliary lithiasis in emergency. Only a simple cholecystectomy & a toilette of the operative field is performed; the endoscopist resolves the choledocholithiasis by a needle sphyncterotomy trailing a catheter introduced into the cystic duct through the papilla. We hypothesize the following results: 1. The reduction of surgical stress & thus the morbidity & mortality rate. 2.The resolution of choledocholithiasis without supra-duodenal or trans-duodenal surgical exploration of the common bile duct. 3.Drainage of the biliary tree without T-tube.4.The reduction of complications resulting from endoscopic sphincterotomy by transpapillary stenting.

### VF015 INTRAHEPATIC LITHIASIS: TREATMENT BY MEANS OF TRANSHEPATIC PERCUTANEOUS ELECTROHYDRAULIC LITHOTRIPSY

G. Tagliabue, E. Faleschini, F. Colturani,
F. Callioni, G. Nervetti and V. Rovati
Cattedra di Patologia Chirurgica
Univ. di Milano

The data in worldwide literature shows that the treatment of intrahepatic lithiasis often requires a combined multidisciplinary approach. The Authors present an example of massive intra and extrahepatic lithiasis treated with three different combined methods:

1) Surgical approach "Cleaning" of the extrahepatic bile duct and of the left intrahepatic bile duct with positioning of the "T" tube

2) Transhepatic percutaneous approach: "cleaning" of the right intrahepatic bile duct by means of an electrohydraulic lithotriptor under colangioscopic control.

3) Endoscopy approach: - papillotomy and collection of the fragment fallen into the main bile duct after lithotripsy. More room is granted to the images illustrating the TRANSHEPATIC PERCUTANEOUS method of LITHOTRIPSY in all its phases:

* Selection of the bile duct to be cannulated
* Preparation of transhepatic percutaneous tramitis by means of dilatators
* Use of the Electroidraulic Lithotriptor under colangioscopic control
* Collection of fragments (Dormia Basket - Fogarthy ballons - Flushing with physiological saline solution).

References:

1) EBBS S.R. et al.:Br.Med.Jour. 292:94 1986

2) MAY R.E.,CORFIELD A.:ANN.R.COLL.SURG.ENGL. 67:96-8 1985

VF016

### PERORAL CHOLEDOCHOSCOPY AND ELECTROHYDRAULIC LITHOTRIPSY FOR LARGE COMMON DUCT STONES

SC Sydney Chung, Joseph WC Leung, Loretta YS So, Arthur KC Li.
Combined Endoscopy Unit, Prince of Wales Hospital, Chinese University of Hong Kong, Shatin, Hong Kong.

With advances in endoscopic techniques and instrumentation it is now possible to directly inspect the bile duct and the pancreatic duct using the endoscopic retrograde route. Direct visual access to the biliary tree opens up new horizons in the diagnosis and therapy of biliary diseases. This video illustrates per-oral choledochoscopy using the "Mother and Baby" endoscope system and electrohydraulic lithotripsy of large common duct stones under direct endoscopic control.

Per-oral choledochoscopy uses two endoscopes. The "Mother" scope (Olympus XTJF-5.5) is a jumbo-sized side viewing duodenoscope with an external diameter of 14.8mm. It has a 5.5mm instrument channel which can admit the "Baby Scope". The "Baby" scope (Olympus CHF-B20) has a working channel of 1.7mm, water and air insufflation, and two way (up-down) control for tip deflection. The procedure is performed under intravenous sedation. The "Mother" scope is first inserted into the duodenum in the short scope position. The "Baby" scope is then passed through the instrument channel of the mother and manipulated into the bile duct (or the pancreatic duct) through a prior endoscopic sphincterotomy. Direct inspection of intra-ductal abnormalities such as tumour or strictures and biopsies of these lesions can be obtained through the "Baby" endoscope.

The technical difficulty of endoscopic stone extraction from the common bile duct increases with the size of the stone. Because the size of a safe endoscopic sphincterotomy is limited, large stones need to be fragmented before endoscopic removal. If the stone can be engaged in a Dormia basket they can be crushed mechanically. Giant stones may be difficult or impossible to engage in baskets because of lack of space in the duct to open the basket. Fragmentation of giant common duct stones using electrohydraulic lithotripsy through the "Mother & Baby" endoscope system is demonstrated. The lithotripsy probe is passed through the channel of the "Baby" scope and pressed against the stone. Because of the risk of duct damage if the probe is fired against the duct wall, direct visual control of electrohydraulic lithotripsy is necessary. A naso-biliary catheter is used to fill the bile duct with saline and to clear away debris generated during lithotripsy. After lithotripsy the stone fragments are removed with Dormia baskets and balloon catheters.

VF017 BILIARY LITHOTRIPSY USING MOBILE LITHOTRIPSY UNIT, DEVELOPMENT OF A MULTICENTRE CONCEPT

J.J.Jakimowicz, F.Schol, Catharina Hospital Eindhoven The Netherlands
G.v.Bergen Henegouwen, H.Obertop, University Hospital Utrecht.

Encouraging results are achieved with biliary extra corporal shockwave Lithotripsy (B-ESWL). Therapy in leading centres result in a successful fragmentation rate up to 98% and when combining B-ESWL with dissolution therapy in 80 to 90% final success is achieved. The answer to the questions whether this therapy has a long standing endresult, whether it is cost efficient and whether the good results are reproducible also out of highly experienced centres should be given. To enable answering these questions we have chosen for a multicentre approach, using mobile Dornier MPL 9000 Lithotripsy unit. The basic consideration of the concept is to enable the patient suffering biliary lithiasis access to a new therapeutic modality in his own region of inhabitants avoiding the necessity of referral, enable physicians to treat their patients at local hospital using the optimal equipment dedicated for this particular application. The mobile Dornier MPL 9000 Lithotripsy unit is presented, the essential technical aspects of the equipment are demonstrated and briefly discussed. An example of treatment using this system is shown and the technical aspects of the procedure as medication, the treatment itself and the dissolution threrapy following Lithotripsy are discussed. The essential points of the protocol study, used by the Worldgroup for gallstone Lithotripsy using a mobile Lithotripsy system, are briefly presented. The preliminary experience, gained in 12 in this project already effectively participating hospitals and the experience with the first 100 patients treated, shows that use of the mobile B-ESWL system fulfils the expectation. The draw-backs limiting spread of a method as limited number of patients in a single hopsital on a year base suitable for B-ESWL and the high costs of dedicated equipment are this way overruled. A multicentre approach offers a unique opportunaty for a porspective study of all the aspects of a method in huge patients population on a long term base.

VF018

### RESECTIVE SURGERY FOR BILE DUCT CYSTS

M.Montorsi, U.Fumagalli,
R.Rosati, M.Zago, A.Poggio and
G.Pezzuoli
1st Surgical Department
University of Milan, Milan, Italy

Bile duct cysts represent an uncommon pathology in the western world. New concepts in pathogenesis which include these cysts in the wider group of the malformations of the bilio-pancreatic ducts and the high complication rates after conservative surgery have made surgeons turn from a mostly derivative approach to a radical resective treatment. The surgical technique for resection of the dilated duct has been standardised in these last years. This video reports the technical aspects in the resective treatment of type III and IV bile duct cysts. During the period 1.1982 - 1.1989, 6 patients with bile duct cysts were observed at the First Surgical Clinic of the University of Milan. According to Todani's classification, 1 patient presented with type Ic cyst, 2 patients with type III, 2 patients with type IVa and 1 patient with type V cysts. The patient with type Ic cyst underwent cholecystectomy and transduodenal sphincterotomy. The other patients underwent resection of the dilated ducts: we performed 2 resections of the choledococele (type III), 2 resections of the extrahepatic bile duct with hepaticojejunostomy at the hilar plate (type IVa) and 1 left lateral segmentectomy with intra-hepatojejunostomy (type V). All patients are well and symptomless at the time of the present report (mean follow up: 41 mos).

A conservative surgery can still be advised only for type Ic fusiform cysts. Radical resection of the extrahepatic bile duct with high bilio-enteric anastomosis is the procedure of choice in the treatment of other type I cysts and for type IV malformations. The prevention of further contact of pancreatic juice with the residual biliary epitelium and the free bile flow should limit the risk of neoplastic changes of the biliary wall. Surgical resection of the choledococele is advised in case of large, symptomatic type III cysts.

### VFO19 SURGICAL TREATMENT OF HYDATID CYSTS OF THE LIVER

G. Romeo, G. Catania, F. Cardì, G. Azzarello,
E. O. Chiarenza, A. Bucceri
Surgical Pathology 2nd - Catania University
Faculty of Medicine, Catania, Italy

Several surgical techniques have been used in recent years for treatment of hydatid cysts of the liver. Conservative surgery implies removal of the contents leaving pericystium in situ; the residual internal cavity is closed by capitonnage, that is by joining the surfaces with interrupted suture in absorbable material, or it is closed by tunnellization onto an external drainage. Demolitive or radical operations include total or subtotal removal of pericystium, sometime associated to some kind of hepatic resection.

Conservative surgery, undoubtedly simpler and low-risk, is however a frequent cause of prolonged post-operative recovery and is followed by high risk of recurrences (about 15-20 per cent), due to failure of pericystium removal and exogenic vesciculation. Radical surgery, although more difficult and with higher operative risk, has better expectancy of complete recovery.

We strongly prefer the radical operations, but in our opinion the choice between the various surgical techniques (total, subtotal or partial pericystectomy) should be established for each patient, after evaluation of several factors: patient's age and general conditions; number, size and topography of cysts and specially their relationship with vasculo-biliary structures.

**Personal experience (1980-89)**

| | | |
|---|---|---|
| Patients operated 56 ( with 72 cysts ) | | |
| Tecniques: cystectomy with partial pericystectomy | | 16 |
| cystectomy with subtotal pericystectomy | | 42 |
| total cistopericystectomy | | 14 |
| Complications: infections | 2 | |
| residual cavity | 3 | |
| recurrent cysts | 0 | |
| Operative and perioperative mortality: 0 | | |
| Mean hospital stay : 11 days (range 8-17) | | |

### VF020 POST TRAUMATIC HEMOBILIA

G.Gozzetti, A.Mazziotti, E.Jovine, R.A.Roversi, G.L.Grazi, A.Frena, U.Grandi, A.Cavallari.

2° Dept. of Surgery, University of Bologna, Italy

The pathophysiological mechanism of hemobilia are discussed on the basis of clinical observations which required different treatment modalities such as direct hemostasis, arterial ligature, liver resection.

The video shows a segmentary liver resection for delayed hemobilia due to a post-traumatic intrahepatic aneurysm. Intraoperative echography was essential in localizing the intrahepatic lesion and in guiding the extent of the resection.

### VF021 HEPATIC CENTRAL BISEGMENTECTOMY FOR ADVANCED GALLBLADDER CANCER

H SHIMADA, K KATAYAMA, G NAKAGAWARA
The First Department of Surgery, Fukui Medical school,
Fukui, JAPAN

The surgical management of gallbladder cancer is influenced considerably by the anatomic spread of tumor and tumor staging.
We will show a film of hepatic central bisegmentectomy for gallbladder cancer with direct invasion of the liver.

The patient was a 73-year-old female complaining of right hypochondric pain. CT showed a clumpy mass in the fundus of the gallbladder extending to Segment IV and V of liver. Endoscopic retrograde cholangiogram showed the obstruction of the cystic duct and narrowing of the common hepatic duct.
Intraoperative echogram showed that the gallbladder mass had expanded to within 2cm on the right of the falciform ligament. The common bile duct was divided at the supraduodenal region. The common hepatic artery, proper hepatic artery, portal vein and supra mesenteric artery were encircled by tapes to dissect en block hepatoduodenal ligament lymphnodes, celiac lymphnodes, retropancreaticoduodenal lymphnodes and lymphnodes around common hepatic artery. After the gallbladder was freed from the liver bed, the anterior branch of the right hepatic artery and portal vein, middle hepatic artery, caudate branch of portal vein and caudate short hepatic veins were divided from below toward the cephalad as far as we could.
After the division of the left hepatic duct and the anterior branch of the right hepatic duct, a central bisegmentectomy was done with total caudate lobectomy by dividing the middle hepatic vein using the CUSA system by extending from the left incision at the sulcus of the falciform ligament to the right side at lobar plane of the demarcation line. Aorto caval lymphnode dissection around the left renal vein was done. A Roux-en-Y loop of jejunum was brought up in a retrocolic fashion and anastomosed to the left hepatic duct.
A histologic examination showed tubular adenocarcinoma of the entire gallbladder with spreading of cancer cells around the cystic duct, including metastasis of the hepatoduodenal and paraaortic lymphnodes.
The patient was discharged 4 months after the operation after receiving chemotherapy because of Virchow lymphnodes.

**VF022 RESECTION OF A HUGE HEPATOCELLULAR CARCINOMA OF SEGMENT ONE (VIDEO FILM)**

D. ELIAS.
Institut Gustave-Roussy (IGR) 94805 Villejuif Cedex, France.

An hepatocellular carcinoma of twelve centimeters of diameter occured on a 68 years old women. It invaded the whole of segment one of the liver, and passed behind the retrohepatic vena cava. It was encapsulated, and the left liver was atrophied secondarly to the compression of the left portal vein. The complete resection was possible by a left hepatectomy associated with complete resection of segment I. We began to expose the posterior face of the portal bifurcation and to cut all the portal veins going to segment I.

During section intermittent clamping of the portal trial was used (it lasted 20 mn plus 20 mn plus 8 mn) and the complete dissection of vena cava and inferior face of the median hepatic vein were necessary.

A triple clamping was performed during 8 Minutes for closing a tear of the anterior face of the vena cava. The operation lasted height hours (one third of the duration was due to the film) and the blood loss was 2.8 liters.

The resection was macroscopically and microscopically curative. The post-operative course was uneventfull and the patient was discharged from hospital on the thirtenth post-operative day.

**VFO23** EXTENDED RIGHT HEPATECTOMY WITH CAUDATE LOBECTOMY FOR HILAR CHOLANGIOCARCINOMA

H SHIMADA, K KATAYAMA, S OHTA, M SAITO, G NAKAGAWARA

The First Department of Surgery, Fukui Medical School, Fukui, Japan

In order to perform the radical resection for hilar cholangiocarcinoma, various modes of hepatic resection as the involvement region are necessary. We will show a film of an extended right hepatectomy with caudate lobectomy for hilar cholangiocarcinoma.

The patient was a 35-year-old male complaining of jaundice. The direct cholangiogram showed the obstruction of the hepatic duct bifurcation and stricturc extending from the common hepatic duct to individual hepatic ducts. The thumb head sized hard tumor was palpated at the hilar region, no hepatic metastasis could be found. The common duct was transected and tied at the supra duodenal region and the right hepatic artery bifurcated from SMA was divided. Then the common bile duct was turned upward. Skeletonization of the hepatoduodenol ligament was completed excluding the arteries and the portal vein.

After exposure of the right branch of the portal vein, it was secured and divided. And the portal branches to the caudate lobe were divided safely due to no vascular involvement by the tumor.

As the right hepatic vein and hepatic short veins across the front of the vena cava were divided, the caudate hepatic veins could be approached safely by adding a direct approach from the lesser sac after division of greater hepatic omentum.

After dividing the left hepatic duct at the bifurcation of the middle lobe branch, several portal middle lobe branches were ligated and divided at the right margin of the ligamentum teres. And extended right lobectomy involving partial resection of S IV was done dividing the middle hepatic vein branches using CUSA and hemoclips.

IORT was done at the surgical margin of the left hepatic duct at a dose of 25 gray.

A Roux-en-Y loop of jejunum was anastomosed to the left hepatic duct using a single layer of interrupted absorbable suture.

Histological examination of the tumor showed tubular adenocarcinoma was located at the hilar region extending to the individual hepatic ducts, with slight hepatic invasion and lymphnode involvement of the hepatoduodenal ligament, retropancreaticoduodenal and celiac regions. This patient was well 4 months after the operation.

### VF024 LIVER TRANSPLANTATION

G. Gozzetti, A. Cavallari, A. Mazziotti, R. Bellusci, E. Jovine, G.L. Grazi, A. Frena, U. Grandi
2° Dept. of Surgery, University of Bologna, Italy

The technique of orthotopic liver transplantation in a woman with primary biliary cirrhosis is presented. A veno-venous by-pass was used during the anhepatic phase.

The vascular anastomoses were performed according to Starzl's technique. Furthermore the usefulness of intraoperative eco-Doppler is stressed in verifying the patency of arterial anastomosis, employing special high resolution probes.

### VF025 A SIMPLE TECHNIQUE OF HEPATIC ARTERY RECONSTRUCTION IN ORTHOTOPIC LIVER TRANSPLANTATION IN THE RAT

R.Steffen, D.Ferguson, R.A.F.Krom,
Mayo Clinic, Rochester MN, USA
H.Keck, B.Lefebre, FU Berlin, FRG

Because of the difficulty of the arterial anastomosis in orthotopic liver transplantation in the rat, usually only the vena cava and vena portae are reconstructed using Kamada's cuff technique. Not reconstructing the hepatic artery leads frequently to bile duct complications jeopardizing the interpretation of the results. We developed a simple technique of arterial reconstruction based on the cuff technique with a patency rate of more than 80% (Steffen 1989). This tape is recorded through the operating microscope and lasts 23 minutes. All donor, back table, and recipient procedures are demonstrated with spoken commentary on important steps. The arterial reconstruction with donor celiac artery and recipient common hepatic artery is shown in detail under high power magnification. The portal and infrahepatic vena cava anastomoses are performed as decribed by Kamada. However, for the suprahepatic vena cava a suture anastomosis using a short donor vena cava segment is demonstrated. An end-to-end bile duct anastomosis is performed by a running suture over a stent. This technique does not require additional skill and does not prolong the procedure significantly. The rat model of orthotopic liver transplantation with arterial reconstruction prevents complications due to bile duct necrosis and is, therefore, particularly ideal for longer term survival studies.

References:

R.Steffen, D.Ferguson, R.A.F.Krom, Transplantation 1989: 48: 166

### VF026 OPERATIVE CHOLEDOCHOSCOPY USING FLEXIBLE CHOLEDOCHOSCOPE

J.J.Jakimowicz, H.Rutten, Catharina Hospital Eindhoven, The Netherlands.

Operative choledochoscopy as a completion procedure after standard instrumental exploration of a common bile duct is generally accepted in the Netherlands. This videotape presents flexible choledochoscope (Olympus Optical Company), discusses the criteria the equipment should fulfil to enable easy instrumentation. The technique of examination using the flexible scope is presented with particular attention to the practical hints, examples of different findings are shown. Choledochoscopy as a completion procedure after exploration of the common bile duct is evaluated based upon 12 years experience using flexible scope. Operative choledochoscopy was performed in 589 patients, 447 patients suffered from choledocholithiasis. Retained stones in the biliary tract missed by routine instrumental exploration, were detected by means of choledochoscopy in 38 patients, 6,5%. Stones retained despite choledochoscopy were found only in 6 patients, 1%. The comparison of personal experience within this series of experience of other surgeons and registrars performing the biliary tract operations led us the conclusion that, if a technique of operative choledochoscopy using flexible scope is well standarized and correctly learned no difference in achieved results is to expect. Operative choledochoscopy is a reliable method of examination supporting valuable diagnostic information contributing to the operative decision making and reducing effectively the incidents of retained biliary stones. It has to be recommanded as a method of choice and as a mandatory completion procedure after performing surgical exploration of a common bile duct.

### VF027 SURGICAL TREATMENT OF PANCREATITIS SECONDARY TO PANCREAS DIVISUM.

EM Targarona, M Garcia, A Gual, C Guevara*, JM Bordas*, E Veloso, C Marco.
Deparment of Surgery and Radiology, Hospital de Mutua de Terrassa.*Endoscopy Unit, Hospital Clinic. University of Barcelona, Barcelona, Spain.

Pancreas divisum (PD) is an anatomical variant of pancreatic ducts that appears in 1-6% of normal population. The relationship between PD and pancreatitis remains controversial. The hypothesis more widely acceppted suggest that the accesory papila, in some cases of PD, may be extremely narrow, and induce obstructive pancreatitis. The demonstration by ultrasonography of a maintained dilatation of the main pancreatic duct after secretin stimulation in patients with PD, supports this hypothesis (1), and this method has been useful in the selection of surgical cases. MATERIAL AND METHODS. From January 1.987 to December 1.989 we have surgically treated 4 patients who presented relapsing pancreatitis secondary to PD, from a consecutive serie of 146 cases of acute pancreatitis (2,7%). This 4 patients are included in a group of 28 with PD, observed in a whole series of 1325 pancreatograms (2,1%) studied by CPRE between 1.979-1.989. Of this 28 cases of PD, only in 14 could be demonstrated pancreatic disease. SURGICAL TREATMENT. 1 patient developed main duct dilatation after 5 episodes of acute pancreatitis, and a pancreatojejunostomy was performed. 3 cases were treated with sphyncteroplasty of the minor papilla, and in one of them, a distal pancreatectomy was added due to the existence of a seudocyst in the tail of the gland. Recurrent symptoms were observed in 1 case, after reestenosis of the sphyncteroplasty. In all the cases, the secretin test observed preoperatively a delay in the empting of the main pancreatic duct, that resolved after dorsal sphyncteroplasty. COMMENT. PD is a variation of the pancreatic duct anatomy, that in few instances may be responsible of acute pancreatitis. This anomaly may be surgically corrected. We present in this video the rationale for the surgical approach of PD, illustrated by the case of a 29 year old women, who presented 3 episodes of acute pancreatitis in the last 5 years. Others causes of pancreatitis were ruled out, except for PD, demonstrated by CPRE. Secretin test was positive. The patient was treated with a sphyncteroplasty of the papilla minor, and remains asymptomatic 1 year after.

1. Warshaw, AL. Am J. Surg. 149:65, 1.985.

### VF028 DUCTAL OCCLUSION IN EXPERIMENTAL ACUTE PANCREATITIS AND ITS POSSIBLE APPLICATION IN PANCREAS TRANSPLANTATION

F Torino, E Deluca, M Garaycoechea
H Garcia, M Viviani, J Bustamante
S G Perera
Churruca Hospital, Buenos Aires
Argentina

An experimental study on a new therapy for acute pancreatitis is presented.

In 88 mongrel dogs the pancreatic ductal system was blockaded after having provoked acute pancreatitis with CaCl; the mortality of this model is 100% within 48 hours. The results were as follows:

| | Series Group | Cases | Death | % |
|---|---|---|---|---|
| 1 | CaCl only (control) | 5 | 5 | 100 |
| 2 | CaCl + Santorini duct ligation | 10 | 10 | 100 |
| | Ductal Occlusion | | | |
| 3 | CaCl + prolamine | 37 | 5 | 16.3 |
| 4 | CaCl + silicone | 10 | 1 | 10.0 |
| 5 | CaCl + fibrin glue | 26 | 3 | 11.5 |

The prolamine and silicone, even though effective, produce an intense fibrosis and atrophy of the lobe. This is not so when fibrin glue is used.

We carried on with the research to find out how and where the ductal blockade develops this therapeutic action. Likewise, new investigations were initiated on the possible application of the ductal blockade with fibrin glue or other reabsorbable substance in the transplant of pancreas. In this way the blockade is temporary and suspends the pancreatic secretion in the critical period after operation without provoking in the gland an irreversible and dangerous fibrosis, perhaps responsible for complications remote from the graft.

References: Torino F et al. Rev Argent Cirug 1983; 44: 31-33
Estourgie R et al. Jour Res. 1983; 34: 164-170
Clemens M et al. XIV Meeting of European Pancreatic Club
Torino F et al. Rev Argent Cirug 1985; 49: 276-279
Gil O et al. Rev Argent Cirug 1986; 50: 1-2 1987; 52: 52-61
Torino F et al. Jour Chir 1989; 126, 2: 88-90

VF029 **SURGICAL TREATMENT OF NECROTIZING PANCREATITIS: NECROSECTOMY AND LAVAGE OF THE LESSER SAC:**

M. Büchler, R. Bittner, R. Roscher,

H.G. Beger

Department of Surgery, University of Ulm, FRG

Necrotizing pancreatitis is still a life threatening disease with mortality rates up to 60%.
The indication for surgery, the timing of surgical intervention and the special type of surgical procedure are the three decisive questions to adequately treat these patients.
In a video (18 minutes) the technique of the Ulm procedure which has led to a considerable reduction of mortality (Beger et al: Br J Surg 1988, 75, 207-212) will be presented.
The video includes perioperative treatment (intensive care etc.), the indication for surgery, the details of surgical handling intraoperatively and the postoperative management of the patient applying continous lavage of the lesser sac and necrotic cavities.

### VF030 LATERO-LATERAL PANCREATOJEJUNAL ANASTOMOSIS IN CHRONIC PANCREATITIS.

A.M.Taschieri, F.Botti, C.Crosta, A.Carrara

F.L.Dassi, M.Nosotti.

Institute of General and Thoracic Surgery
Milan, Italy

McCaugham in 1938 and Cattell in 1947 first suggested pancreato-jejunal derivation as the treatment for pancreatic pain originating from hypertension of the ductal system.
In 1960, Partington and Rochelle proposed their own technique, which consisted in an ample longitudinal opening of Wirsung's duct and its anastomosis to a retrocolic Roux en Y loop with the end towards the tail of the pancreas to minimise angulation.
Partington and Rochelle's operation revealed a number of advantages: it was easy to perform, the morbidity and mortality were truly negligible, no orgarn was demolished and it gave excellent, immediate results.
We have applied this procedure in cases of chronic pancreatitis in generally alcoholic patients with painful reactions to medical treatment and with a Wirsung's duct dilatated to over 7 mm.
The results we have obtained in our Institute are encouraging: there has been no morbidity or mortality and pain controls two years after the operation are good to fair in 90% of patients.
In the videotape we present our own technical devices on latero-lateral pancreato-jejunal anastomosis.

References:

E.L.Bradley Am.J.Surg.1987; 153: 207-213
Ph.Morel, A.Rohner Surgery 1987; 101,2: 130-135
P.F.Partington, R.E.L.Rochelle Ann.Surg.1960; 152,6: 1037-1043

**VFO31 PANCREATIC ASCITES: SURGICAL MANAGEMENT.**

L. FERNANDEZ-CRUZ, J.M. LLOVERA,
A. SAENZ, E. ASTUDILLO, X. PIULACHS,
E.M. TARGARONA.
**UNIVERSITY OF BARCELONA. HOSPITAL CLINIC. DEPT. OF SURGERY. BARCELONA, SPAIN.**

Five patients with internal pancreatic fistula and pancreatic ascites are reported. Diagnosis was made by finding a markedly elevated amylase and protein content in the ascites fluid. All patients had history of inflamatory pancreatic disease.

In the video is shown the case of a patient with pancreatic ascites and jaundice in whom surgical therapy was available. ERCP showed multiple pancreatic pseudocyst and stones in the distal end of the common bile duct. Upon surgical exploration leakage of pancreatic secretions into the peritoneal cavity was seen. Peroperative Wirsungography confirmed the presence of multiple pancreatic pseudocyst. 80 % pancreatic resection was performed and the pancreatic remanent was anastomosed to a Roux-en-Y loop (pancreatico-yeyeunostomy). The head of the pancreas was enlarged and indurated and a large fluctuant pseudocyst was found. The pseudocyst was drained to de Roux-en-Y loop (cysto-yeyunostomy). The hepatic duct was also anstomosed to the yeyeunum (hepatico-yeyeunostomy).

The purpose of this video is to review the optimal diagnostic approach and essential principles of surgical treatment of pancreatic ascites.

**VFO32** MICROSURGERY APPLIED TO THE EXPERIMENTAL PANCREATIC TRANSPLANTATION

L.LORENTE, J.ARIAS, J.RODRIGUEZ,

J.I.TROBO, A.CABALLERO, E.TEJERO,

H.DURAN. Hospital San Carlos, Madrid

SPAIN

Pancreatic transplantation is an efective treatment of insulin-dependent diabetes in humans, althought the inherent complications of the surgical technique still require the experimental development of variants capable of securing a low percentage of complications.

A microsurgical technique for the autotransplantation of the pancreas in the dog is improved on. In group A (n=6) paratopic autotransplantation with portal venous drainage of the graft. The main pancreatic vessels were anastomosed to the hiliary branches of the splenic vessels. Exocrine secretion was free within the peritoneal cavity. In group B (n=9) heterotopic autotransplantation with systemic venous drainage of the graft. The main pancreatic vessels were anastomosed to the lingual artery, and to the linguál facial venous trunk. The pancreatic duct was anastomosed to the bucal mucosa. In both groups, four to six weeks after autotransplant, a right pancreatectomy was performed. There wasn't graft atrophy and the graft exocrine functionalism to the bucal cavity was verified during postoperative evolution. Endocrine performance was determined by the intavenous glucose tolerance test during six months postoperative.

The results show taht vascular microsurgery applied to the segmentary autotransplantation of the pancreas in the dog avoid thrombosis of the graft and facilitates longterm survival of the animal. Segmental transplantation could act by substituing the metabolic needs in insulin-dependent diabetes mellitus, where the secondary risk of thrombosis possibly disappears provided revascularization is carried out respecting pancreatic tail and body characteristics.

**VF033 EXOCRINE AND ENDOCRINE FUNCTION OF SEGMENTAL PANCREATIC AUTOTRANSPLANTATION AFTER SUBTOTAL PANCREATIC RESECTION FOR CHRONIC PANCREATITIS.**

L. FERNANDEZ-CRUZ, E. ASTUDILLO,
A. SAENZ, J.M.LLOVERA, M.LOPEZ-BOADO
M. PERA, G. BENARROCH.
**UNIVERSITY OF BARCELONA. HOSPITAL CLINIC. DEPT. OF SURGERY. BARCELONA, SPAIN.**

Segmental pancreatic autotransplantation has been performed to prevent the severe metabolic complications of extended pancreatic resection. The major problem with segmental pancreatic graft relates the handling of the pancreatic duct and its secretion. In this video we present two patients with intraperitoneal (vascular anastomosis to the iliac vessels) segmental pancreatic autotransplantation anastomosed to a Roux-en-Y loop after subtotal pancreatectomy for chronic pancreatitis.

Physiologic studies indicate normal exocrine and endocrine function 1 year following transplant. The patients are insulin-independent and tolerates a normal meal, requering no oral pancreatic enzyme supplementation.

This technique should be considered as an alternative for there patients who require extensive resection for chronic pancreatitis.

